# Facile Preparation of Ultrafine Porous Copper Powders for Accelerating the Thermal Decomposition of Ammonium Perchlorate

**DOI:** 10.3390/ma17235728

**Published:** 2024-11-23

**Authors:** Dayong Li, Yuling Shao, Shengquan Chang, Yanggang Huang, Yong Kou, Lei Xiao, Gazi Hao

**Affiliations:** 1 China North Chemical Research Institute Group Co., Ltd., Beijing 100089, China; shaoyuling19870903@163.com; 2 Liaoning Qingyang Special Chemical Co., Ltd., Liaoyang 111000, China; changshengquan@163.com; 3 National Special Superfine Powder Engineering Research Center of China, School of Chemistry and Chemical Engineering, Nanjing University of Science and Technology, Nanjing 210094, China; 13736564171@163.com (Y.H.); kouyong0313@163.com (Y.K.); 15005161138@163.com (L.X.)

**Keywords:** Cu powders, ammonium perchlorate, chemical reduction, catalytic properties, thermal analysis

## Abstract

In this study, we innovatively proposed a facile method to synthesize ultrafine porous copper (Cu) powders under mild conditions by utilizing the reduction properties of reduced iron (Fe) powders. The results showed that Cu^2+^ was easily reduced to Cu at 1.05–1.1 times the theoretical iron powder content for a reaction time of 10~20 min at 20~25 °C. The obtained Cu powders with an average diameter of 10.2 μm did not show significant differences in crystal structure and purity compared to the commercial Cu powders with an average diameter of 6.6 μm, but the prepared Cu powders showed a loose and porous structure, which demonstrates their higher potential in catalyzing energetic materials. The ultrafine porous Cu powder resulted in a significant decrease in the high decomposition temperature of ammonium perchlorate (AP) from 441.3 °C to 364.2 °C at only 1% of the dosage, and also slightly advanced its low decomposition temperature, which confirmed its remarkable catalytic activity in the field of energetic materials. These meaningful results will provide a new method for the preparation of Cu powders and promote the development of the chemical reduction method for the preparation of ultrafine porous Cu powders, which is expected to promote the application of ultrafine porous Cu powders in the field of energetic materials catalysis.

## 1. Introduction

The synthesis and application of catalysts have always received a lot of attention from researchers as they allow for significant catalytic effects to be achieved from very small amounts. In recent years, the synthesis of copper (Cu) powders has rapidly advanced with the development of basic science and preparation technology [1,2,3]. Ultrafine Cu powders with appropriate microstructures are widely used in various fields, such as metallurgy, electrode materials, conductive coatings, lubricants, machining, and catalysis [4,5,6,7,8,9]. Among various transition metal catalysts (Fe, Ni, Cu, etc.), Cu exerts a remarkable catalytic effect on the thermal decomposition of energetic materials [10]. The main methods of preparing Cu powder for industrial production are electrolysis and atomization; both methods have their own characteristics [11,12,13,14,15,16,17]. The electrolytic method has many advantages, such as high purity, a large specific surface area, a low apparent density, and good compressibility and molding. However, it suffers from some drawbacks, including high energy consumption, high cost, serious pollution of the environment and insufficient yield. The atomization approach is widely used to produce a wide range of semi-spherical Cu powders [18,19]. Unfortunately, it involves a series of complex processes, such as atomization, oxidation and reduction, and grinding, resulting in increased time and costs. Therefore, it is of great value to search for simple and available methods for industrial preparation of ultrafine Cu powders with high purity and yield.

Fortunately, the chemical reduction method has attracted growing attention due to its unique advantages, such as a short process time, the simple equipment used, low cost, and its convenience for industrial preparation. Additionally, it can be utilized to prepare high-performance catalysts [20,21,22,23]. However, a large number of reducing agents, such as formaldehyde, sodium hypophosphite, and hydrazine hydrate, are used in the chemical reduction method, which causes a large amount of damage to the environment due to its high toxicity and greatly restricts its widespread use [24,25]. To address the above bottlenecks, our group proposes a more efficient and environmentally friendly chemical reduction technology for the preparation of Cu powder based on the reduction properties of reduced Fe powder (α-Fe, Cubic). The reduced Fe powder is synthesized by reducing Fe oxide with carbon materials and then heat-treating it in a hydrogen atmosphere, which provides it with a wide range of applications as an available source and clean reducing agent in powder metallurgy, as well as other physical and chemical applications [26,27,28,29,30]. The preparation of reduced Fe powder, as well as the new preparation technology for Cu powder using reduced Fe powder as a reducing agent, shows great promise due to the high-efficiency, green, and safety properties of the raw material, as well as the reduction process.

Catalysts are commonly used in propellants to improve the thermal decomposition and combustion properties of oxidizers (e.g., ammonium perchlorate (AP), ammonium nitrate (AN), ammonium dinitramide (ADN)), so as to significantly enhance the overall performance of the propellants [31,32,33,34,35,36]. Among them, AP is the most widely used oxidizer in composite solid propellants, and it is very beneficial for the oxidation and combustion of the other components as it has the highest oxygen balance (+34.04%). Its higher hydrogen content also contributes to its energy conversion efficiency [31,37]. In addition, AP also has the characteristics of low mechanical sensitivity and good thermal and chemical stability, and the proportion of AP in the propellant is as high as 60~80%, which greatly affects the performance of the propellant [38,39]. However, the unconcentrated exotherm of AP due to its two decomposition stages greatly reduces its energy-release efficiency. It is of great significance to improve the performance of the propellant by promoting the centralized heat release of AP. Apparently, the burning rate catalysts are able to accelerate the thermal decomposition process of AP, and copper salts exhibit an excellent catalytic ability regarding the thermal decomposition of AP [40,41,42,43,44,45]. Interestingly, some studies have also proposed that metals are more efficient catalysts compared to metal oxides. The metal reacts with the oxygenated substances produced during decomposition to form metal oxides in the presence of a large amount of heat, thus exhibiting a stronger catalytic activity than the metal oxide particles [46]. The porous structure endows Cu powder with a superior catalytic ability due to its larger specific surface area and more active sites. The ultrafine porous Cu powders prepared in this study are expected to exhibit remarkable catalytic effects on AP. Herein, we report the facile preparation of ultrafine porous Cu powders with a loose and porous microstructure using the chemical reduction method with reduced Fe powders as a reducing agent. Furthermore, the catalytic properties of the obtained ultrafine porous Cu powders on the thermal decomposition process of AP were also investigated and were also compared with the catalytic properties of commercial Cu powders. The results indicate that the novel preparation technique for ultrafine porous Cu powders utilizing reduced Fe powder as a reducing agent could be widely used in the field of energetic materials catalysis to greatly improve the properties of energetic materials.

## 2. Materials and Methods

### 2.1. Materials

Copper sulfate pentahydrate (CuSO_4_·5H_2_O, 99.0%), Commercial Cu Powder, reduced iron powder (Fe ≥ 98%, *d*_50_ = 34 μm), sulfuric acid (H_2_SO_4_, ~98%) and ethyl acetate (CH_3_COOC_2_H_5_, 99.0%) were purchased from Sinopharm Chemical Reagent Co., Ltd. (Shanghai, China) (The reduced iron powders have the following composition, %: ≥98 Fe; ≤0.1 insoluble in H_2_SO_4_; ≤0.06 sulfide; ≤0.005 total nitrogen (N); 0.03 substances soluble in water; ≤0.005 Cu.) AP (chemical grade, *d*_50_ = 64 μm) was brought from Dalian North Potassium Chlorate Co., Ltd. (Dalian, China). All chemicals were used as received without further purification.

### 2.2. Preparation of Ultrafine Porous Cu Powders

Firstly, 40 g of CuSO_4_·5H_2_O was dissolved in 1 L of deionized water to form a blue solution with continuous stirring. Secondly, a few drops of sulfuric acid were added to the above solution to adjust the pH to close to 2.0. Thirdly, 9.4~9.8 g of Fe powder (1.05~1.1 times the theoretical dosage of Fe powder) was added to the above blue solution and stirred for 10~20 min at different temperatures at 20~25 °C. The aqueous solution was cleared to form a deep red-colored Cu powder. Next, the product was separated by filtration and washed several times with deionized water, and then dried under vacuum at 50~80 °C for 1~2 h. Finally, the products were ground in a mortar with a very weak force, and then purified by magnetic separation to remove trace amounts of residual Fe so as to produce Cu powders with pure, fluffy, ultrafine, and porous characteristics.

### 2.3. Preparation of the AP/Cu Mixture

The AP/Cu mixture was prepared using the grinding method. Firstly, 0.01 g of Cu powder and 0.99 g of AP were added to 3 mL of ethyl acetate and dispersed ultrasonically for 5 min. Secondly, the mixture was transferred to an onyx mortar and lightly ground to promote the adequate mixing of AP and Cu powder. Finally, after ethyl acetate evaporation, the mixture was dried in a drying oven at 50 °C for 10 min to obtain the AP/Cu mixture. The mixtures of AP with 1% of prepared Cu powder and AP with 1% of commercial Cu powder are marked as M1 and M2, respectively.

### 2.4. Characterization

X-ray powder diffraction (XRD) patterns collected on a Bruker D8-Advanced diffractometer with Cu Kα1 radiation (λ = 0.15406 nm) were applied to analyze the crystal structure of the products. The morphology of Cu powders was characterized using a field emission scanning electron microscope (FE-SEM, Hitachi S-4800 II from Hitachi) (Tokyo, Japan), and their chemical compositions were determined via energy dispersion X-ray spectrometer (EDS). The chemical compositions of two different Cu powders were also analyzed using a PerkinElmer Optima 7000 DV elemental analyzer from PerkinElmer. (Shelton, CT, USA) Thermogravimetric (TG) analysis was also carried out to determine the purity of obtained Cu powders at a heating rate of 20 °C min^−1^ from 50 to 800 °C with an air flow rate of 20 mL/min. The particle size distribution of samples was detected using a laser diffraction technique with a Mastersizer 2000 analyzer from Malvern Panalytical Ltd, (Malvern, UK). For comparison, commercial Cu powders with an average diameter of 6.6 μm were also measured. PHdz-01 of pen type pH meter (Resolution: 0.01 pH), which is produced by Shanghai dapu Instrument Co., Ltd. (Shanghai, China), was used to accurately measure the acidity (pH) of the aqueous solution. Apparent density was determined using a calibrated funnel.

### 2.5. Measurement of Catalytic Properties

The thermogravimetric (TG) analysis and differential scanning calorimetric (DSC) techniques were applied to investigate the thermal decomposition of raw AP as well as the AP/Cu mixtures so as to probe the catalytic properties of the prepared Cu powders. The thermal decomposition properties of raw material AP and AP/Cu mixtures were tested on an SDT Q600 differential scanning calorimeter form TA Instruments (New Castle, Delaware, DE, USA) at 50~500 °C with heating rates of 5, 10, 15, and 20 °C·min^−1^ and a nitrogen flow rate of 20 mL/min. The kinetic data of the mixtures were calculated according to the corresponding DSC data to reveal the remarkable catalytic properties of the prepared Cu powders.

## 3. Results

### 3.1. Formation Process of Ultrafine Porous Cu Powder

The preparation of Cu powder using chemical reduction method with reduced Fe powder as the reducing agent uses green and environmentally friendly technology, as no toxic substances are used or produced during the preparation process. Although the reduction of Cu powder seems to be a simple process, it involves a variety of chemical reactions, as shown in Equations (1)–(7) [47]. First, Cu^2+^ undergoes an obvious substitution reaction with Fe upon contact, as in Equation (1). Secondly, Fe^3+^ is reduced to Fe^2+^ under slightly acidic solution conditions, as in Equation (2) [48]. Equations (2) and (5) are weak reactions in weakly acidic solutions, where Fe^3+^ has a greater oxidizing power than Cu^2+^. Fe is oxidized to Fe^2+^ under acidic conditions (as in Equation (3)) but is oxidized to Fe^3+^ in the presence of O_2_ (as in Equation (6)), and Cu exhibits the same behavior (as in Equation (4)). It is worth noting that Equation (7) is unavoidable due to the very small *K*_sp_ of Fe(OH)_3_. The pH of the solution should be lowered to prevent the formation of Fe(OH)_3_ precipitates during the preparation of the Cu powder. The maximum concentration of Fe^3+^ has been reported to reach 156 ppm at pH = 2, so it should be adjusted to 2 [49]. When the pH in solution is 2, the main reaction is Equation (1); reaction processes such as Equations (2), (5) and (7) can be attenuated. The reaction process such as Equation (3) is very weak and unavoidable, and it has little effect on the main reaction. The reaction processes such as Equations (4) and (6) can be ignored in deionized water. In addition, an excess of the reducing agent can promote a more complete reaction and avoid the formation of Fe^3+^.

(1) $${\mathrm{Fe}+\mathrm{CuSO}}_{4}\to {\mathrm{Cu}+\mathrm{FeSO}}_{4}$$

(2) $${\mathrm{Fe}+2\mathrm{Fe}}^{3+}\to 3{\mathrm{Fe}}^{2+}$$

(3) $${\mathrm{Fe}+H}_{2}{\mathrm{SO}}_{4}\to {\mathrm{FeSO}}_{4}+H_{2}↑$$

(4) $${2\mathrm{Cu}+2H}_{2}{\mathrm{SO}}_{4}{+O}_{2}\to {2\mathrm{CuSO}}_{4}{+2H}_{2}O$$

(5) $${\mathrm{Cu}+2\mathrm{Fe}}^{3+}\to {\mathrm{Cu}}^{2+}+{2\mathrm{Fe}}^{2+}$$

(6) $${4\mathrm{FeSO}}_{4}{+2H}_{2}{\mathrm{SO}}_{4}{+O}_{2}\to {2\mathrm{Fe}}_{2}{{(\mathrm{SO}}_{4})}_{3}{+2H}_{2}O$$

(7) $${\mathrm{Fe}}^{3+}+3H_{2}O\to {\mathrm{Fe}(\mathrm{OH})}_{3}↓+3H^{+}$$

**Figure 1:**
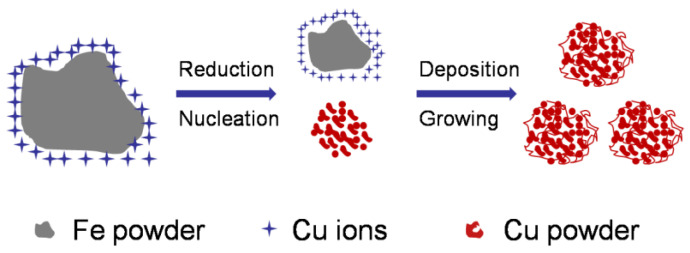
Illustration of the synthesis of ultrafine porous Cu powders via chemical reduction.

It has been reported that the replacement reaction between Fe and Cu is controlled via diffusion through the mass transfer boundary layer, and the reaction follows first-order kinetics [47,50]. Apparently, a large amount of Cu^2+^ will be encapsulated in Fe powder when reduced Fe powder is added to the Cu^2+^ solution, and the generated Cu will transfer to the solution via diffusion, producing nuclei and consequently leading to the growth of Cu powder. Meanwhile, the loose and porous structure of Cu powder will be formed with its own deposition characteristics and the presence of H_2_ [51,52,53]. The formation process of Cu powder with a loose porous structure is shown in Figure 1.

Furthermore, the effects of preparation conditions such as the content of the reduced Fe powder, the reaction temperature, the reaction time and the pH of solution on the yield of Cu powder were investigated, and the results are shown in Table 1. The results show a significant increase in the yield of Cu powder with the increase in Fe powder content during the reaction process. The increase in reaction temperature and reaction time had no remarkable effect on the yield of Cu powder, but the increase in the pH of the solution significantly decreased the yield of Cu powder. The yield of Cu powder reached up to 99.8% under optimal conditions, confirming the great potential of preparing Cu powder using this method.

### 3.2. The Morphology and Structure of Ultrafine Porous Cu Powders

**Figure 2:**
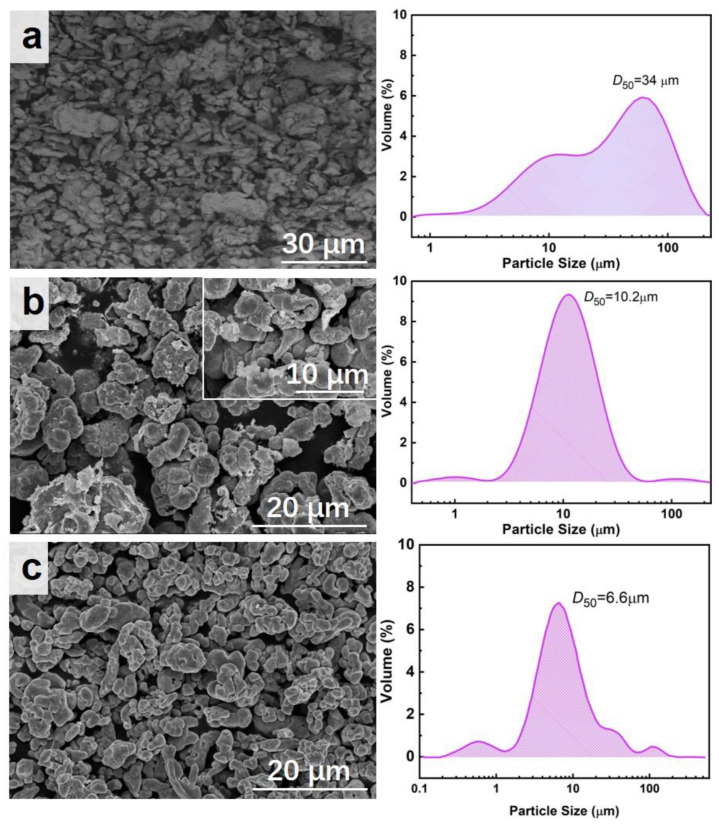
SEM and particle size of different samples: (**a**) the reduced Fe powders; (**b**) the obtained Cu powders; (**c**) commercial Cu powders.

The microscopic morphology and particle size of the reduced Fe powder, prepared Cu powder, and commercial Cu powder are shown in Figure 2. Figure 2a clearly shows the irregular microscopic morphology of reduced Fe powder with an average particle size of 34 μm but a wide particle size distribution of 1~150 μm. Figure 2b demonstrates that the prepared Cu powder has a spherical-like morphology as well as a narrow particle size distribution, with an average particle size of 10.2 μm. The local enlarged image of Figure 2b clearly reveals the porous structure of the prepared Cu powder, which is more favorable for the catalytic performance due to its richer active sites. As a comparison, the microscopic morphology and particle size of commercial Cu powder are shown in Figure 2c. The result shows the spherical shape of the commercial Cu powder with an average particle size of 6.6 μm. The microstructure of Cu powder is the key factor affecting its catalytic performance; that is, different structures will lead to distinct differences in its catalytic performance. The apparent density of the prepared Cu powders is 1.932 g·cm^−3^, while that of the commercial Cu powders is 3.296 g·cm^−3^. The larger particle size of the Cu powders leads to a smaller apparent density, further confirming their porous structure.

**Figure 3:**
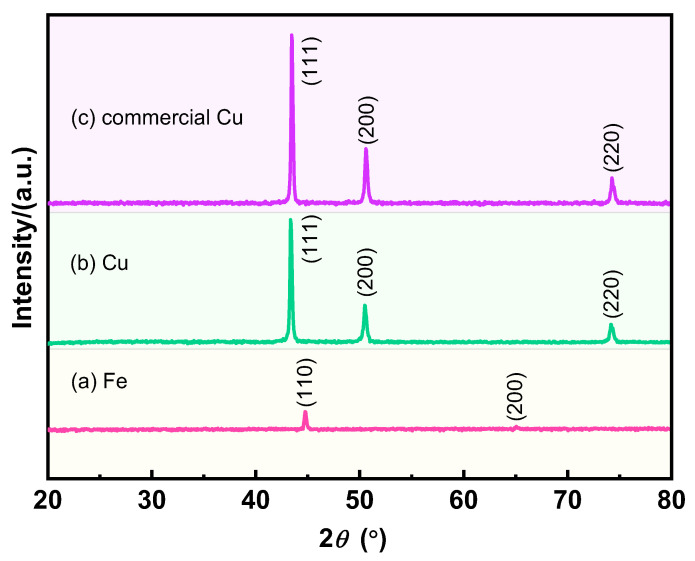
XRD patterns of different samples: (a) the reduced Fe powders; (b) the obtained Cu powders; (c) commercial Cu powders.

The crystal structures of the different samples were further explored, and the results are shown in Figure 3. The two diffraction peaks of the reduced Fe powder belong to the (110) and (200) crystal planes, which correspond to the standard data (JCPDS 65-4899), proving its body-centered cubic structure (α-Fe) without impurities. The prepared Cu powders and commercial Cu powders exhibit the same three diffraction peaks, which correspond to the (111), (200), and (220) crystal planes, respectively, indicating that the prepared porous Cu powders and the commercial Cu powders are face-centered cubic structures (JCPDS 65-9026). The similar crystal structures of the prepared Cu powders and commercial Cu powders confirm the high purity of the ultrafine porous Cu powders and also demonstrate the feasibility of the preparation method.

**Figure 4:**
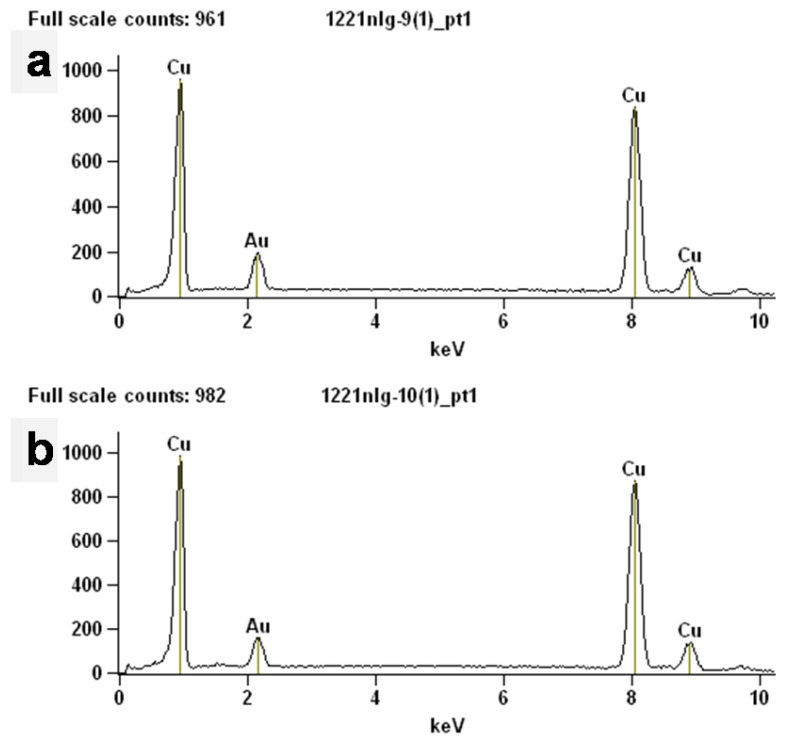
EDS spectra of different samples: (**a**) the obtained Cu powders; (**b**) commercial Cu powders.

The elements of the prepared Cu powder and commercial Cu powder were tested using EDS to analyze their purity, and the results are shown in Figure 4. The results of the EDS show that both the prepared Cu powder and commercial Cu consisted of pure Cu, which indicates the high purity of the prepared Cu powder and is in agreement with the results of the XRD. Furthermore, the chemical compositions of two different Cu powders were detected using an elemental analyzer to confirm the high purity of the prepared Cu powders, and the results are shown in Table 2. The two Cu powders both have high purity, with the purity of the prepared Cu powders reaching as high as 99.785%, which corresponds to the results of the EDS. This conclusion further confirms the great advantage of the reduction method for the preparation of Cu powders.

**Figure 5:**
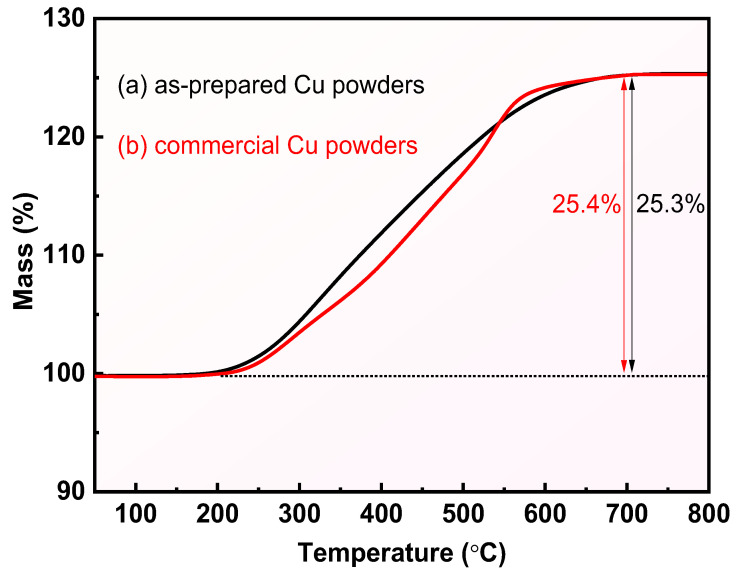
TG curves of (a) the obtained Cu powders and (b) commercial Cu powders.

In addition, the thermal oxidation process of the prepared Cu powder and commercial Cu powder in the range of 50–800 °C was explored using TG, and the results are shown in Figure 5. This Figure clearly shows that both the prepared Cu powder and commercial Cu powder exhibit a significant mass increase in the range of 250~600 °C. The percentage mass of the prepared Cu powders and commercial Cu powders increased by 25.3% and 25.4%, respectively, which indicates that both Cu powders were completely converted to CuO according to the theoretical mass change of 25.2% for the conversion of Cu to CuO. The above results reconfirm the high purity of the prepared Cu powders as well as the great potential of the reduction method for the preparation of Cu powders.

### 3.3. Catalytic Properties of Ultrafine Porous Cu Powders

**Figure 6:**
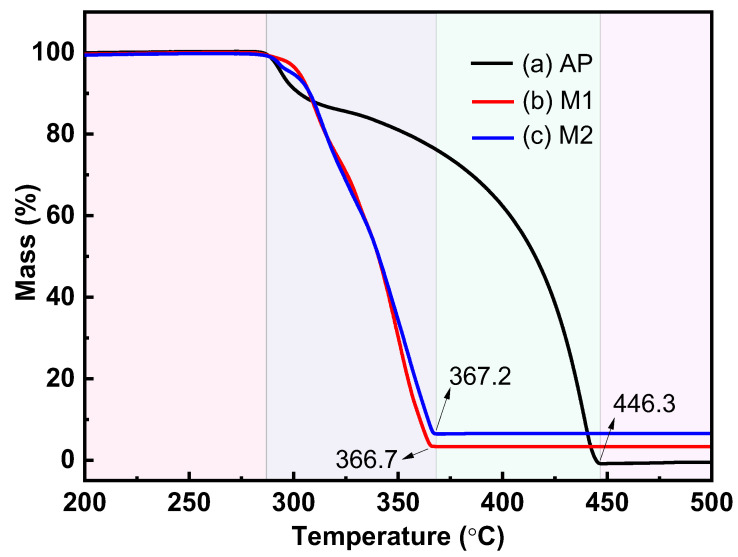
TG curves for the thermal decomposition of different samples: (a) raw AP; (b) M1; (c) M2.

**Figure 7:**
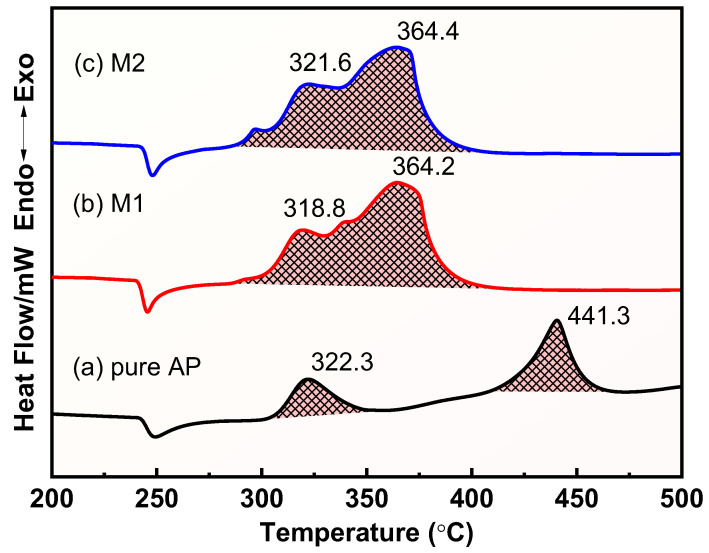
DSC curves for the thermal decomposition of different samples: (a) raw AP; (b) M1; (c) M2.

TG-DSC tests were carried out on raw AP as well as AP mixtures to explore the catalytic properties of the prepared Cu powders and commercial Cu powders, and the results are shown in Figure 6 and Figure 7. Figure 6 clearly shows two distinct weight loss stages of the raw AP, which correspond to the low-temperature decomposition stage and the high-temperature decomposition stage of the AP, respectively. The prepared Cu powders, as well as the commercial Cu powders, allow for an almost overlap in the thermal decomposition stages for AP, and the end temperatures of the thermal decomposition of M1 and M2 are reduced from 446.3 °C to 366.7 °C and 367.2 °C, respectively, showing a favorable catalytic effect.

The DSC curves in Figure 7 further show the thermal decomposition process of the different samples. The raw AP has an obvious absorption peak at 247 °C, which is caused by the phase transition of AP from orthorhombic to cubic, and there is no obvious mass loss in this process [53]. The exothermic peaks located at 322.3 °C and 441.3 °C corresponded to the low-temperature decomposition stage and high-temperature decomposition stage of AP, respectively, consistent with the results of TG. The thermal decomposition process of AP is complex, including a coupled solid phase, adsorbed phase, and gas phase reaction, and the thermal decomposition mechanism of AP is still not fully understood. There are several thermal decomposition mechanisms of AP; the proton transfer mechanism proposed by Jacobs has been supported by most of the research [31]. During the low-temperature decomposition process, the protons are transferred from NH_4_^+^ to ClO_4_^−^ to generate gaseous NH_3_ and HClO_4_, which are adsorbed on the surface of AP to prevent its further decomposition. AP undergoes a partial decomposition during this stage. The low-temperature decomposition process is shown in Equation (8). The high-temperature decomposition stage mainly involves the desorption of NH_3_ and its oxidation by the decomposition products of HClO_4_, which releases a large amount of heat that is fully converted to gaseous products.

(8) $${\mathrm{NH}}_{4}^{+}{+\mathrm{ClO}}_{4}^{-}{=\mathrm{NH}}_{3}{(a)+\mathrm{HClO}}_{4}{(a)=\mathrm{NH}}_{3}{(g)+\mathrm{HClO}}_{4}\left(g\right)$$

Obvious heat absorption peaks also appeared near 247 °C in mixtures M1 and M2, indicating that the presence of the two Cu powders did not change the phase transition process of AP. The high decomposition temperatures of M1 and M2 decreased from 441.3 °C to 364.2 °C and 364.4 °C when the content of Cu powder was 1%, a decrease of 77.1 °C and 76.9 °C, respectively, showing the significant catalytic effect of Cu powder on the thermal decomposition process of AP, especially the prepared Cu powder. The correlation data of the DSC curves for AP, M1, and M2 are shown in Table 3. The 1% prepared Cu powders and commercial Cu powders increased the apparent decomposition heat of M1 and M2 from 941 J/g to 1598 J/g and 1587 J/g at the heat rate of 20 °C·min^−1^, respectively, an increase of 69.8% and 68.7%, respectively, confirming the significant catalytic effect of Cu powders on the thermal decomposition process of AP. The catalytic ability of a catalyst decreases with an increase in its particle size, but although the prepared Cu powder has a larger particle size than that of commercial Cu powder, it shows a better catalytic performance than that of commercial Cu powder, which further confirms the great advantage of the reduction method for the preparation of Cu powder. The efficient catalytic effect of the prepared Cu powders is attributed to their porous structure and high purity, which confers more active sites, thereby improving its catalytic activity compared to commercial Cu powders.

**Figure 8:**
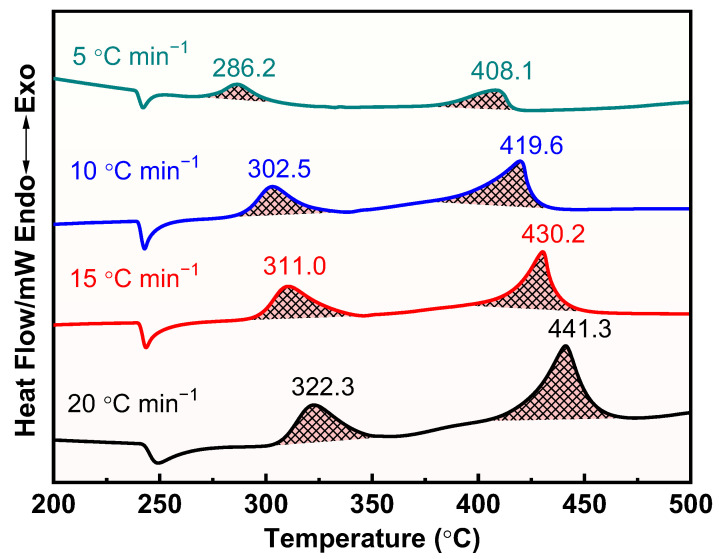
DSC curves for the decomposition of pure AP at different heating rates.

**Figure 9:**
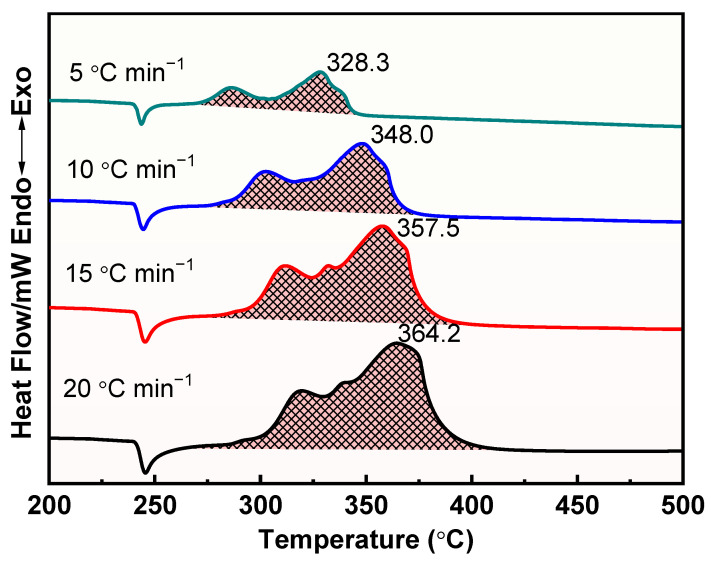
DSC curves for the decomposition of M1 at different heating rates.

**Figure 10:**
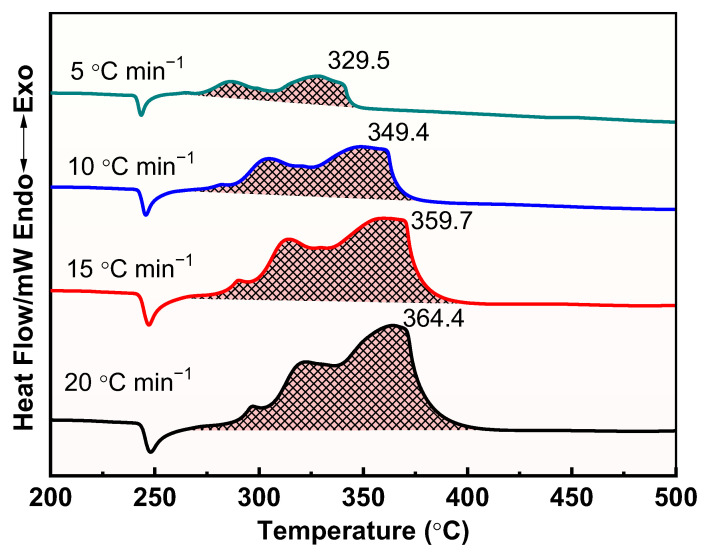
DSC curves for the decomposition of M2 at different heating rates.

The thermal decomposition process of raw AP, M1, and M2 at different heating rates was further investigated, and the results are shown in Figure 8, Figure 9 and Figure 10. Apparently, the thermal decomposition temperature of the samples shows a significant hysteresis effect with increasing heating rate, which is due to the uneven heating of the samples caused by the accelerated heating rate. Meanwhile, the thermal decomposition stages of M1 and M2 were merged into one, exhibiting a more concentrated exotherm with the addition of prepared Cu powder and commercial Cu powder, which is attributed to the efficient catalytic effect of Cu powders, especially the prepared Cu.

In addition, three methods can be used, those of Kissinger, Ozawa, and Starink, to calculate the thermal decomposition kinetic parameters for different samples, as shown in Equation (9) [54]. The Kissinger method was used to calculate the DSC thermal decomposition parameters for AP, M1, and M2, due to its characteristics of rapidity, simplicity, and reliability [55]. At the same time, the frequency factors and reaction rate constants were calculated using the Arrhenius equation, as shown in Equation (10).

(9) $$\ln\left(\frac{\beta }{T_{p}^{s}}\right)=-B\frac{E_{a}}{RT_{p}}+\mathrm{constant}$$

where *β* is the heating rate (°C min^−1^), *T*_p_ is the peak temperature (*T*_H_), R is the ideal gas constant, *E*_a_ is the activation energy, *s* is a constant, and *B* is a constant that depends on the choice of s. In the case of the Kissinger method, *s* = 2 and *B* = 1, for the Ozawa method, *s* = 0 and *B* = 1.0518, and for the Starink method, *s* = 1.8 and *B* = 1.0070–1.2 × 10^−5^ *E*_a_ (*E*_a_ in kJ·mol^−1^).

(10) $$k=A\cdot \exp\left(-\frac{E_{a}}{RT_{p}}\right)$$

where *A* is the frequency factor, while the *β* is 20 °C min^−1^, *k* is the reaction rate constant, and *T* is equal to *T*_p_.

The thermal decomposition kinetic parameters of AP, M1, and M2 during the high-temperature decomposition stage are shown in Table 4. Compared with the raw AP, the *E*_a_ of M1 and M2 decreased from 141.8 kJ/mol to 111.8 kJ/mol and 112.7 kJ/mol in the presence of 1% of prepared Cu powders and commercial Cu powders, respectively, decreases of about 30.0 kJ/mol and 29.1 kJ/mol compared to raw AP, indicating the better thermal decomposition properties of M1 and M2.

It is worth noting that the *E*_a_ of M1 is slightly lower than that of M2, and the relationship between *E*_a_ and conversion (*α*) was further explored to analyze the difference between the *E*_a_ of M1 and M2. The Friedman isoconversion method is widely used to calculate the relationship between *E*_a_ and conversion rate α. The α was calculated using TG data (Figure S1), as in Equation (11).

(11) $$\alpha =\frac{m_{0}-m_{t}}{m_{0}-m_{f}}$$

where *m*_0_, *m_t_*, and *m_f_* are the masses at the start, *t*, and end moments, respectively.

The Friedman isoconversion method is shown in Equation (12).

(12) $$\ln(\frac{d\alpha }{dT}\beta )=-\frac{E_{a}}{RT}+\ln(A(f(\alpha )))$$

**Figure 11:**
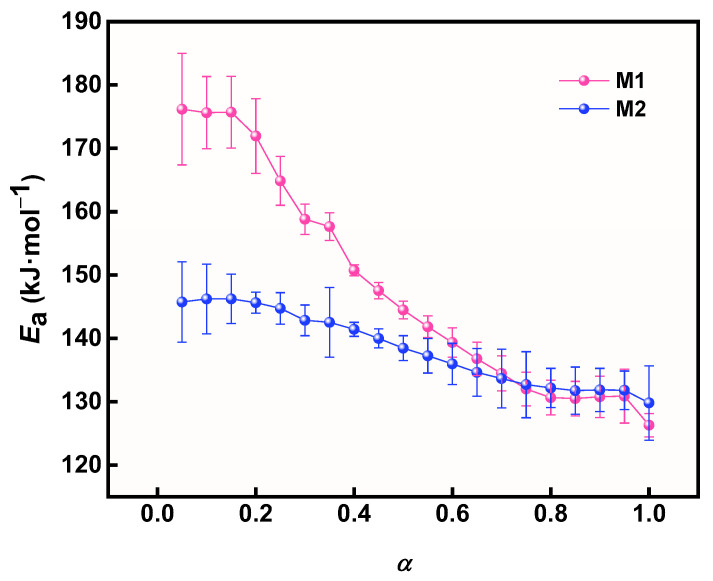
The relationship between activation energy and α of different samples.

The relationship between *E_a_* and *α* during the thermal decomposition of M1 and M2 is shown in Figure 11. The variation in *E_a_* with *α* for M1 and M2 shows a significant difference. This clearly shows that the *E_a_* of M1 and M2 gradually decreases with increasing *α*, especially M1. Specifically, the *E_a_* value of M1 decreased significantly more than that of M2 when *α* was increased from 0.05 to 1, demonstrating the significant catalytic effect of prepared Cu powder on AP.

**Figure 12:**
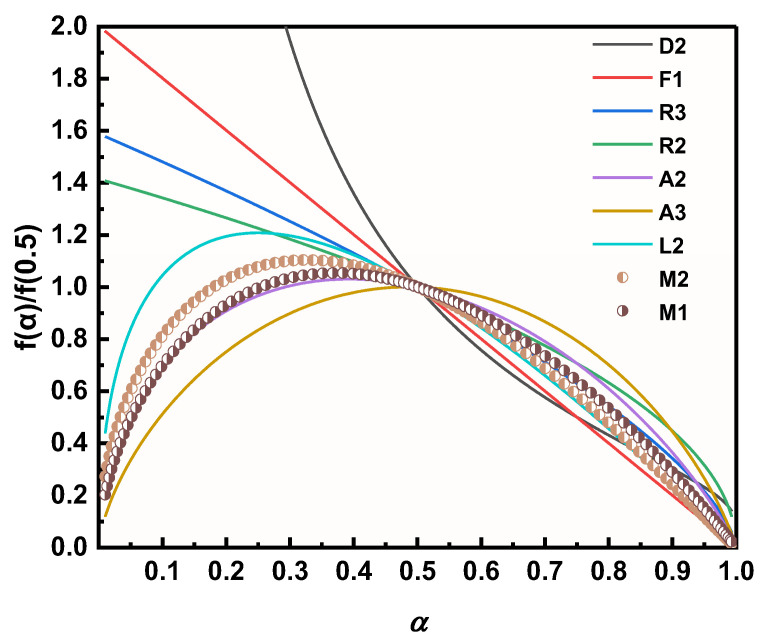
Thermal decomposition kinetic modeling of different samples.

The decomposition kinetic models of M1 and M2 in the presence of two Cu powders were further explored using a combined kinetic analysis method (CKA method) (as in Equation (13)), so as to investigate the effect of different Cu powders on the thermal decomposition process of AP, and the results are shown in Figure 12 [56]. The results clearly show that the decomposition model of M2 is between A2 and L2 and closer to the A2 model, while the decomposition process of M1 perfectly fits the A2 decomposition model, which implies that the thermal decomposition process of M1 is more in line with the two-dimensional nuclear growth model after stochastic nucleation [57].

(13) $$\ln(\beta \frac{d\alpha }{dT{\left(1-\alpha \right)}^{n}\alpha^{m}})=-\frac{E_{a}}{RT}+\ln(CA)$$

The above results demonstrate the excellent catalytic activity of the prepared ultrafine porous Cu powders on the thermal decomposition of AP. The exothermic peak temperature and reaction activation energy of AP can be remarkably decreased and the apparent decomposition heat, as well as the reaction rate constant of AP, can be significantly increased. The porous structure of ultrafine porous Cu powders prepared via the chemical reduction method endow them with more active sites, thereby leading to a better or equivalent catalytic activity than commercial Cu powders.

## 4. Conclusions

In this study, ultrafine porous Cu powders were synthesized using a simple chemical reduction method. This new method of preparing Cu powder has the advantages of requiring a short time and producing a high yield. The obtained Cu powders were characterized by their high purity, better catalytic properties than commercial copper powders, and loose and porous structure, with an average particle size of 10.2 μm. The encouraging results are very attractive for the development of a green, low-energy, and high-efficiency process for the preparation of ultrafine porous Cu powders, thus suggesting the application of ultrafine porous Cu powders in the accelerated thermal decomposition of AP. In subsequent studies, we could further control the particle size of the reduced Cu powders by modulating the experimental parameters to realize the efficient preparation of nano-Cu powders.

## Figures and Tables

**Table 1:** Synthesis conditions and yield of Cu powder.

No.	Dosage of Fe ^a^	Reaction Temperature/°C	Reaction Time/min	Initial pH	Yield/%
1	1	20	15	2	96.3
2	1.05	20	15	2	99.7
3	1.08	20	15	2	99.8
4	1.11	20	15	2	99.8
5	1.05	25	15	2	99.8
6	1.05	25	10	2	99.8
7	1.05	25	15	2.5	99.4
8	1.05	25	15	3	98.5

^a^: The times based on the theoretical amount of Fe powder.

**Table 2:** Chemical compositions of two different Cu powders.

Samples	Cu/%	Fe/%	Pb/%	P/%	Others
prepared Cu powders	99.785	0.012	0.046	0.099	0.058
commercial Cu powders	99.763	0.004	0.088	0.097	0.048

**Table 3:** DSC data of AP, M1 and M2.

Samples	*T*_L_/°C	*T*_H_/°C	*H*/(J g^−1^)	Δ*H*/(J g^−1^)	GR/%
AP	322.3	441.3	941	——	——
M1	323.4	364.2	1598	657	69.8
M2	323.4	364.4	1587	646	68.7

Annotate: *H*—the apparent decomposition heat. Δ*H*—the change in *H* based on AP. GR—the growth rate of H based on AP.

**Table 4:** The kinetic parameters for the HTD of different AP samples.

Samples	*E*_a_ (kJ mol^−1^)	*A* (min^−1^)	*k* (s^−1^)
AP	141.8	1.40 × 10^10^	9.97 × 10^−3^
M1	111.8	9.30 × 10^8^	1.06 × 10^−2^
M2	112.7	1.07 × 10^9^	1.04 × 10^−2^

## Data Availability

The original contributions presented in the study are included in the article and Supplementary Materials, further inquiries can be directed to the corresponding authors.

## Supplementary Materials

The following supporting information can be downloaded at: https://www.mdpi.com/article/10.3390/ma17235728/s1, Figure S1. TG curves of different samples: (a) M1; (b) M2.
