# Supplementary material for: Facile Preparation of Ultrafine Porous Copper Powders for Accelerating the Thermal Decomposition of Ammonium Perchlorate
Source: Materials (Basel). 2024 Nov 23;17(23):5728. doi: 10.3390/ma17235728 (PMC11642433; doi:10.3390/ma17235728)
Supplement: Supplementary file 1 [file materials-17-05728-s001.zip › materials-3291675-supplementary.pdf]

Facile Preparation of Ultrafine Porous Copper Powders for Accelerating the Thermal Decomposition of Ammonium Perchlorate

Dayong Li[1*], Yuling Shao[1], Shengquan Chang[2], Yanggang Huang[3], Yong Kou[3], Lei Xiao[3], Gazi Hao[3*]

1 China North Chemical Research Institute Group Co., Ltd. Beijing 100000, China;

2 Liaoning Qingyang Special Chemical Co., Ltd. Liaoyang 111000, China;

3 National Special Superfine Powder Engineering Research Center of China, School of Chemistry and Chemical Engineering, Nanjing University of Science and Technology, Nanjing 210094, China;

Corresponding author: 43070531@qq.com (Dayong Li); hgznjust1989@163.com (Gazi Hao)

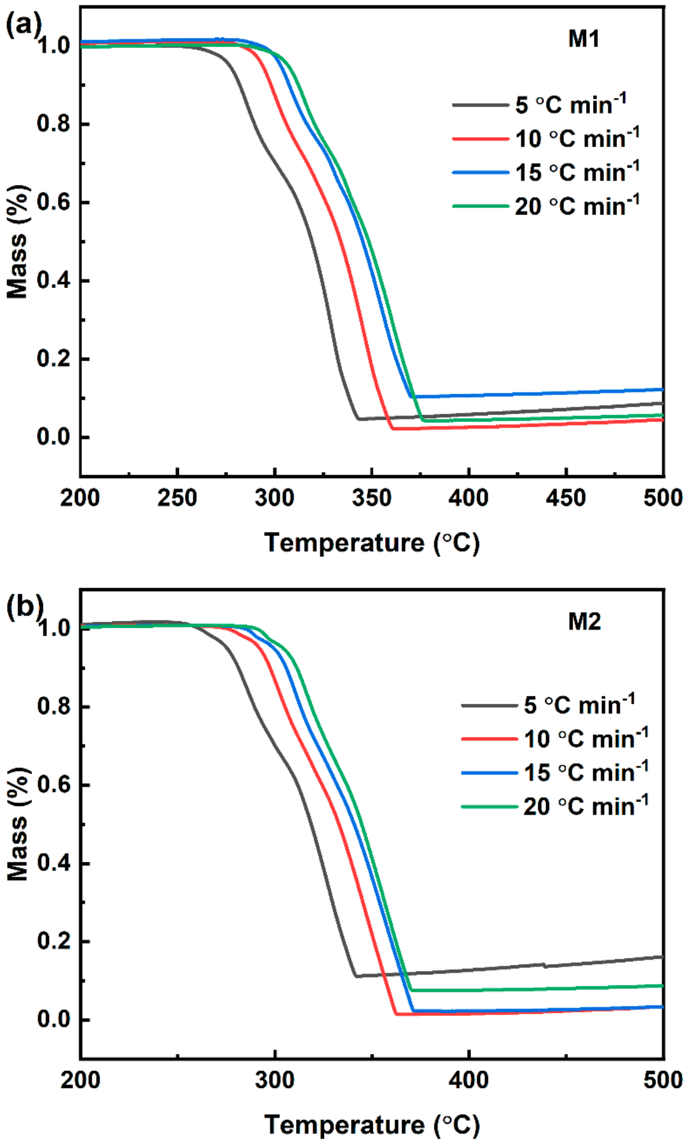

Fig. S1 TG curves of different samples: (a) M1; (b) M2
